# Are ankylosing spondylitis, psoriatic arthritis and undifferentiated spondyloarthritis associated with an increased risk of cardiovascular events? A prospective nationwide population-based cohort study

**DOI:** 10.1186/s13075-017-1315-z

**Published:** 2017-05-18

**Authors:** Karin Bengtsson, Helena Forsblad-d’Elia, Elisabeth Lie, Eva Klingberg, Mats Dehlin, Sofia Exarchou, Ulf Lindström, Johan Askling, Lennart T. H. Jacobsson

**Affiliations:** 1 Department of Rheumatology and Inflammation Research, Institute of Medicine, Sahlgrenska Academy at University of Gothenburg, Box 480, 405 30 Gothenburg, Sweden; 2 Departments of Public Health and Clinical Medicine, Rheumatology, Umeå University, 901 87 Umeå, Sweden; 3 Section of Rheumatology, Department of Clinical Sciences, Malmö, Lund University, 202 13 Malmö, Sweden; 4 Clinical Epidemiology Unit and Rheumatology Unit, Department of Medicine Solna, Karolinska Institutet, 171 77 Solna, Sweden

**Keywords:** Ankylosing spondylitis, Psoriatic arthritis, Undifferentiated spondyloarthritis, Spondyloarthritis, Spondylarthropathies, Cardiovascular disease, Cohort, Acute coronary syndrome, Stroke, Venous thromboembolism

## Abstract

**Background:**

To investigate the risk of first-time acute coronary syndrome (ACS), stroke and venous thromboembolism (VTE) in patients with ankylosing spondylitis (AS), psoriatic arthritis (PsA) and undifferentiated spondyloarthritis (uSpA), compared to each other and to the general population (GP).

**Methods:**

This is a prospective nationwide cohort study. Cohorts with AS (n = 6448), PsA (n = 16,063) and uSpA (n = 5190) patients and a GP (n = 266,435) cohort, were identified 2001–2009 in the Swedish National Patient and Population registers. The follow-up began 1 January 2006, or 6 months after the first registered spondyloarthritis (SpA) diagnosis thereafter, and ended at ACS/stroke/VTE event, death, emigration or 31 December 2012. Crude and age- and sex-standardized incidence rates (SIRs) and hazard ratios (HRs) were calculated for incident ACS, stroke or VTE, respectively.

**Results:**

Standardized to the GP cohort, SIRs for ACS were 4.3, 5.4 and 4.7 events per 1000 person-years at risk in the AS, PsA and uSpA cohort, respectively, compared to 3.2 in the GP cohort. SIRs for stroke were 5.4, 5.9 and 5.7 events per 1000 person-years at risk in the AS, PsA and uSpA cohort compared to 4.7 in the GP cohort. Corresponding SIRs for VTE were 3.6, 3.2 and 3.5 events per 1000 person-years at risk compared to 2.2 in the GP cohort. Age-and sex-adjusted HRs (95% CI) for ACS events were significantly increased in AS (1.54 (1.31–1.82)), PsA (1.76 (1.59–1.95)) and uSpA (1.36 (1.05–1.76)) compared to GP. Age-adjusted HRs for ACS was significantly decreased in female AS patients (0.59 (0.37–0.97)) compared to female PsA patients. Age-and sex-adjusted HRs for stroke events were significantly increased in AS (1.25 (1.06–1.48)) and PsA (1.34 (1.22–1.48)), and nonsignificantly increased in uSpA (1.16 (0.91–1.47)) compared to GP. For VTE the age-and sex-adjusted HRs for AS, PsA and uSpA were equally and significantly increased with about 50% compared to GP.

**Conclusions:**

Patients with AS, PsA and uSpA are at increased risk for ACS and stroke events, which emphasizes the importance of identification of and intervention against cardiovascular risk factors in SpA patients. Increased alertness for VTE is warranted in patients with SpA.

**Electronic supplementary material:**

The online version of this article (doi:10.1186/s13075-017-1315-z) contains supplementary material, which is available to authorized users.

## Background

Spondyloarthritis (SpA) is a group of rheumatic diseases with a spectrum of well-defined clinical features, which include inflammatory back pain, inflammation of the sacroiliac joints, peripheral synovitis, enthesitis, psoriasis, inflammatory bowel disease and anterior uveitis [1].

In contrast to rheumatoid arthritis (RA), cardiovascular risk in SpA is less investigated. The risk of cardiovascular disease (CVD) in SpA was raised as a gap of knowledge in the recently updated European League Against Rheumatism (EULAR) recommendations for CVD management in patients with RA and other forms of inflammatory joint disorders [2]. Studies have indicated that ankylosing spondylitis (AS) is associated with an increased risk of cardiovascular morbidity and mortality, but the results have been divergent [3–10]. Psoriasis has been associated with an increased risk of stroke, myocardial infarction as well as venous thromboembolism (VTE) [11–16]. A study of non-fatal CVD in women with psoriasis found that women with concomitant psoriatic arthritis (PsA) had particularly high risk of non-fatal CVD, which is in line with studies focusing on PsA where an increased risk of CVD has been shown [17–19]. In addition, a higher burden of traditional CVD risk factors as well as increased subclinical atherosclerosis have been observed in AS and PsA patients compared to controls [10, 20–28].

We have previously compared the occurrence of different cardiovascular phenotypes in RA and AS. We found that AS patients had a 30–50% increased risk of incident cardiovascular events compared to general population. When compared to RA patients the level of increase was similar for stroke, but only half as high for acute coronary syndrome (ACS) and venous thromboembolic events [9]. The aim of this study was to extend these assessments of risks for defined cardiovascular phenotypes across different SpA subtypes, and specifically to compare risks in AS, PsA, and undifferentiated SpA (uSpA) to each other and to the general population.

## Methods

### Study design and register sources

This is a prospective nationwide population-based cohort study, including three separate cohorts of patients with AS, PsA, and uSpA identified from the Swedish National Patient Register (NPR) and one general population (GP) cohort identified from the Swedish Population Register. Through the unique personal identification number of each Swedish resident, the subjects were linked to the NPR, the Population Register, Swedish Rheumatology Quality Register (SRQ), the Swedish Prescribed Drug Register (PDR), and Statistics Sweden to identify cardiovascular outcomes as well as sociodemographic data, comorbidities and pharmacological treatment in order to characterize the populations.

The NPR was established in 1964 and contains data of inpatient care, from 1987 with full national coverage. Non-primary outpatient care has been included in the register from 2001. Primary and secondary diagnoses reported by the physician are recorded at each visit according to the International Classification of Diseases (ICD).

The Swedish Population Register holds information of residency, immigration, emigration and deaths for all the Swedish residents.

The PDR started July 2005 and contains information regarding dispensed prescriptions of pharmacological treatment, registered according to the Anatomical Therapeutic Chemical Classification (ATC) system.

The SRQ started in 1995 and includes information on treatment with biological disease-modifying antirheumatic drugs (bDMARDs) from 1999.

The research was performed with the ethical approval of the Regional Ethics Committee, Stockholm, Sweden, and has been carried out in compliance with the Helsinki declaration. Informed consent was not needed due to the register-based study design.

### Study populations

This study is part of a large-scale nationwide register linkage project, which identified all patients with a registered diagnosis of AS, PsA or uSpA, given at a visit to a physician, in the NPR from 1968 through 2009. From the Population Register up to five controls per patient were identified, matched on birth-year, sex and place of residence at the date the index patient received the first SpA diagnosis in the NPR.

### AS, PsA, and uSpA cohorts

From these larger groups of SpA patients, we selected all patients with at least one physician visit with an AS, PsA or uSpA diagnosis in rheumatology or internal medicine outpatient care between 2001 and 2009, at the ages 18 to 99 years, alive and living in Sweden at start of follow-up. These patients were included in either of the AS (*n* = 6448), PsA (*n* = 16,063) or uSpA (*n* = 5190) cohorts. Patients with a diagnosis of RA or systemic lupus erythematosus in rheumatology or internal medicine outpatient care between 2001 and 2009 were excluded.

The ICD codes used in the identification process are shown in Additional file 1. The ICD codes for AS, PsA, and uSpA have previously been validated and shown to have high positive predictive values (PPVs) when compared to established classification criteria [29, 30]. In patients with AS and available data of imaging and/or HLA-B27 status, the PPVs for fulfilling the modified New York criteria and any set of SpA criteria were 80% and 97%, respectively. The corresponding PPVs in uSpA patients were 26% and 89%, respectively. Undifferentiated SpA encompassed both axial SpA as well as peripheral SpA [29]. The PPVs of an ICD code for PsA within primary or specialized care was within the range of 63% to 92%, where the highest proportion of confirmed PsA were noted if the PsA was diagnosed in specialized care (as the PsA patients in the present study) and at least on two occasions [30].

#### Patients with ≥ 2 subtypes of SpA

Patients diagnosed with more than one of the three studied SpA subtypes before start of follow-up were not included in the AS, PsA, and uSpA cohorts but analyzed separately to avoid case mixing (*n* = 1082).

### General population cohort

All initially identified GP controls from the first register extraction, at the ages 18 to 99 years, alive and living in Sweden at start of follow-up, were included in the GP cohort (n = 266,435) and used as unmatched GP comparator subjects. GP comparator subjects were excluded from the GP cohort if they were identified in any of the SpA cohorts before start of follow-up (*n* = 340).

### Follow-up

The follow-up began 1 January 2006 for subjects identified prior to 30 June 2005. For subjects identified later than 30 June 2005, the follow-up began 6 months after the date of the first registered SpA diagnosis in rheumatology or internal medicine outpatient care. We chose 1 January 2006 to identify contemporary cohorts and to enable use of data from the PDR (which started July 2005). The lag period of 6 months was used to avoid detection bias, with the cardiovascular outcome of interest being a consequence of increased surveillance in connection with the SpA diagnosis. For GP comparators the follow-up began 1 January 2006 or the date of immigration if this occurred later than 1 January 2006.

All subjects were followed until first occurrence of cardiovascular outcome, emigration, death or 31 December 2012, whichever occurred first. Subjects were censored in the GP cohort if diagnosed with a SpA diagnosis and subsequently eligible to enter the corresponding SpA cohort. Patients were censored from their original SpA cohort if diagnosed with a second SpA subtype during follow-up and subsequently eligible to enter the SpA cohort with ≥ 2 subtypes of SpA (described above).

### Baseline characteristics

SpA-related comorbidities (anterior uveitis, psoriasis, and inflammatory bowel disease), cardiovascular comorbidities (ischemic heart disease, stroke, VTE, atrial fibrillation/flutter, other atherosclerotic disease), diabetes and chronic obstructive pulmonary disease (COPD) were regarded as prevalent at baseline if the specified ICD codes (see Additional file 1) were identified in the NPR before start of follow-up. Pharmacological treatment at baseline was defined as a dispensed prescription within 6 months before start of follow-up according to specified ATC codes in the PDR (Additional file 1). We also linked data from SRQ for the intravenous TNF inhibitor infliximab since infliximab is administered in a hospital setting and therefore not registered in the PDR. From the Statistics Sweden, we retrieved the highest level of education as a measure of socioeconomic status.

### Definition of cardiovascular outcome

The primary outcomes were incident ACS, stroke, and VTE. Each outcome was analyzed separately. The primary outcomes were identified in NPR and defined as follows:

1. First occurrence of ACS (including acute myocardial infarction and unstable angina), reported as a primary discharge diagnosis from inpatient care.
2. First occurrence of stroke, reported as a primary or secondary discharge diagnosis from inpatient care. In the primary analysis we used a composite stroke outcome, consisting of both ischemic, hemorrhagic, unspecified stroke and transient ischemic attack (TIA). As secondary outcomes ischemic stroke, hemorrhagic stroke and TIA were analyzed separately.
3. First occurrence of VTE reported as a primary or secondary diagnosis from either inpatient or outpatient care.

The ICD codes used to identify the outcomes are described in Additional file 1. Importantly, subjects with a history of the cardiovascular outcome of interest prior to start of follow-up were excluded from that specific analysis. Such exclusion was based on both primary and secondary diagnoses in outpatient and inpatient care.

### Statistical analyses

Descriptive statistics are presented as number (percentage) or mean ± standard deviation (SD). For each cardiovascular outcome incidence rates, overall and stratified by sex, were calculated from the number of incident cardiovascular events and person-years at risk. To enable comparison between the cohorts, standardized rates were calculated, using the age and sex distribution in the GP cohort as standard. For the risk assessment age- and sex-adjusted hazard ratios (HRs) were calculated using Cox proportional hazard regression analyses. The largest SpA cohort – the PsA cohort – was used as the reference population in the comparison between the SpA cohorts. The proportional hazards assumption for the Cox regression analyses were evaluated graphically in survival curves and was considered fulfilled in all cases. Where applicable, 95% confidence intervals (CI) are reported. For incidence rates we have assumed a Poisson distribution when estimating 95% CI. Additionally, age- and sex-standardized prevalence and corresponding prevalence ratio (PR) with 95% CI for prior ACS, stroke, and VTE before start of follow-up were calculated for each SpA cohort, using the age and sex distribution in the GP cohort as standard/reference. Statistical analyses were performed by PASW Statistics version 19 (SPSS Inc., Chicago, IL, USA) and SAS Version 9.3 (SAS Institute Inc., Cary, NC, USA).

## Results

### Study population

We identified cohorts of AS (*n* = 6448), PsA (*n* = 16,063) and uSpA (*n* = 5190) patients and one with GP comparators (*n* = 266,435). Baseline characteristics are described in Table 1. As expected, the AS cohort had a larger proportion of men compared to the other cohorts. In the PsA cohort 41.5% had current disease-modifying antirheumatic drugs (DMARDs) at baseline compared to 24.8% and 30.4% in the AS and uSpA cohort, respectively. Approximately 50% in all of the SpA cohorts were exposed to nonsteroidal anti-inflammatory drugs (NSAIDs) at baseline. The uSpA cohort had the youngest mean age and had overall lower frequencies of previous cardiovascular comorbidity and dispensed prescription of drugs related to CVD. Age- and sex-standardized PRs of prior ACS, stroke, and VTE were increased in all of the SpA cohorts compared to the GP cohort (Additional file 2). The subjects with a prior ACS, stroke and VTE at baseline were excluded from the ACS, stroke and VTE outcome analysis, respectively.

**Table 1:** Baseline characteristics of patients with AS, PsA, uSpA and GP comparators at start of follow-up

	AS (*n* = 6448)	PsA (*n* = 16,063)	uSpA (*n* = 5190)	GP (*n* = 266,435)
Sex				
Males	4390 (68.1)	7217 (44.9)	2325 (44.8)	131,807 (49.5)
Females	2058 (31.9)	8846 (55.1)	2865 (55.2)	134,628 (50.5)
Age (years)				
Mean (SD)	50.0 (13.9)	53.2 (13.8)	44.7 (13.3)	53.5 (15.9)
18–29 years	488 (7.6)	761 (4.7)	691 (13.3)	18,465 (6.9)
30–39 years	1120 (17.4)	2047 (12.7)	1255 (24.2)	36,805 (13.8)
40–49 years	1452 (22.5)	3368 (21.0)	1406 (27.1)	52,122 (19.6)
50–59 years	1670 (25.9)	4385 (27.3)	1085 (20.9)	63,559 (23.9)
60–69 years	1209 (18.8)	3638 (22.6)	583 (11.2)	52,626 (19.8)
70–79 years	414 (6.4)	1422 (8.9)	132 (2.5)	27,259 (10.2)
≥ 80 years	95 (1.5)	442 (2.8)	38 (0.7)	15,599 (5.9)
Start of follow-up				
2006–2007	4938 (76.6)	11,926 (74.2)	3725 (71.8)	266,234 (99.9)
2008–2010	1510 (23.4)	4137 (25.8)	1465 (28.2)	201 (0.1)
Level of education				
-≤9 years	1423 (22.1)	4039 (25.1)	804 (15.5)	68,776 (25.8)
-10–12 years	3096 (48.0)	7983 (49.7)	2566 (49.4)	119,681 (44.9)
->12 years	1858 (28.8)	3931 (24.5)	1789 (34.5)	74,587 (28.0)
-Missing	71 (1.1)	110 (0.7)	31 (0.6)	3391 (1.3)
SpA-related comorbidities^a^				
Anterior uveitis	1342 (20.8)	252 (1.6)	771 (14.9)	1156 (0.4)
Inflammatory bowel disease	514 (8.0)	346 (2.2)	269 (5.2)	2565 (1.0)
Psoriasis	145 (2.2)	15,873 (98.8)	162 (3.1)	2190 (0.8)
SpA-related medications^b^				
Any DMARD	1599 (24.8)	6659 (41.5)	1579 (30.4)	2542 (1.0)
-TNF inhibitors	545 (8.5)	758 (4.7)	279 (5.4)	268 (0.1)
-Methotrexate	494 (7.7)	5094 (31.7)	609 (11.7)	1104 (0.4)
-Sulfasalazin	798 (12.4)	1396 (8.7)	877 (16.9)	493 (0.2)
NSAIDs	3543 (54.9)	7801 (48.6)	2804 (54.0)	28,467 (10.7)
Prednisone	659 (10.2)	2133 (13.3)	778 (15.0)	4234 (1.6)
Other comorbidities^a^				
Ischemic heart disease	468 (7.3)	1091 (6.8)	182 (3.5)	14,849 (5.6)
-ACS	256 (4.0)	576 (3.6)	94 (1.8)	8714 (3.3)
Composite stroke	201 (3.1)	527 (3.3)	84 (1.6)	8954 (3.4)
-Ischemic stroke	92 (1.4)	265 (1.6)	49 (0.9)	5009 (1.9)
-Hemorrhagic stroke	41 (0.6)	100 (0.6)	14 (0.3)	1412 (0.5)
-TIA	77 (1.2)	185 (1.2)	28 (0.5)	2951 (1.1)
Venous thromboembolism	115 (1.8)	347 (2.2)	80 (1.5)	3835 (1.4)
Diabetes	311 (4.8)	1025 (6.4)	144 (2.8)	10,118 (3.8)
COPD	122 (1.9)	317 (2.0)	48 (0.9)	3507 (1.3)
Atrial fibrillation or flutter	264 (4.1)	513 (3.2)	87 (1.7)	7430 (2.8)
Other atherosclerotic disease	210 (3.3)	581 (3.6)	101 (1.9)	8318 (3.1)
Dispensed prescriptions^b^				
Oral antidiabetics or insulin	274 (4.2)	1077 (6.7)	123 (2.4)	11,372 (4.3)
Antihypertensive	1794 (27.8)	4853 (30.2)	846 (16.3)	59,053 (22.2)
Statins	657 (10.2)	1871 (11.6)	307 (5.9)	23,180 (8.7)
Aspirin	557 (8.6)	1519 (9.5)	230 (4.4)	23,655 (8.9)
Warfarin	174 (2.7)	277 (1.7)	52 (1.0)	4328 (1.6)

Age are given in mean (SD). All other data are presented in number (%)

*AS* ankylosing spondylitis, *PsA* psoriatic arthritis, *uSpA* undifferentiated spondyloarthritis, *GP* general population, *DMARD* disease-modifying antirheumatic drug, *NSAIDs* nonsteroidal anti-inflammatory drugs, *ACS* acute coronary syndrome, *TIA* transient ischemic attack, *COPD* chronic obstructive pulmonary disease

^a^Prevalent comorbidity at baseline, defined by identification of specified ICD codes in the National Patient Register prior to start of follow-up

^b^Dispensed prescription in Prescribed Drug Register or intravenous bDMARDs in Swedish Rheumatology Quality register within 6 months prior to start of follow-up

The mean overall time of follow-up (SD) was 5.6 (1.9) years for AS patients, 5.7 (1.6) years for PsA patients, 5.4 (2.0) years for uSpA patients, and 6.7 (1.2) years for GP comparators.

### Acute coronary syndrome

**Fig. 1:**
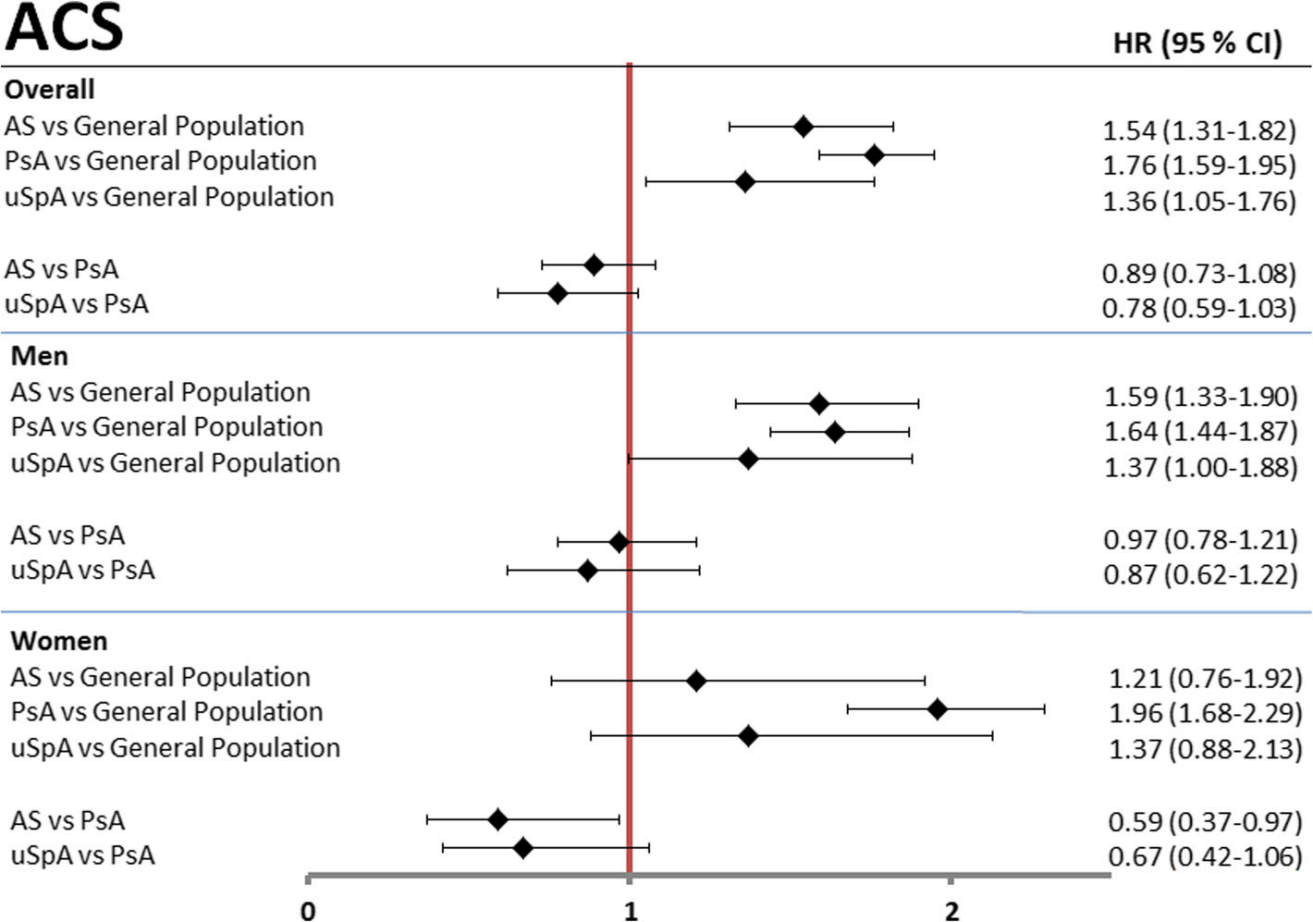
Age- and sex-adjusted hazard ratios (HRs) for acute coronary syndrome (ACS). Age- and sex-adjusted HRs, overall and stratified by sex, are presented with 95% confidence interval (CI) in patients with AS, PsA, and uSpA, using GP comparators and PsA patients as reference. *AS* ankylosing spondylitis, *PsA* psoriatic arthritis, *uSpA* undifferentiated spondyloarthritis

During follow-up, 143, 420, and 59 incident ACS events occurred in the AS, PsA, and uSpA cohorts, respectively, resulting in standardized incidence rates of 4.3, 5.4, and 4.7 ACS events per 1000 person-years at risk compared to 3.2 in the GP cohort (Table 2). The incidence rates were overall lower for women than men. The age- and sex-adjusted HRs were significantly increased in AS (1.54 (1.31–1.82)), PsA (1.76 (1.59–1.95), and uSpA (1.36 (1.05–1.76)) compared to the GP cohort. In the sex-stratified analyses, the highest age-adjusted HR point estimate was noted for female PsA patients (1.96 (1.68–2.29)) compared to the female GP cohort. Female PsA patients also had an increased risk of ACS compared to female AS patients (Fig. 1).

**Table 2:** Incidence rates of ACS, stroke and VTE in AS, PsA, uSpA patients and GP comparators

	AS	PsA	uSpA	GP
Acute coronary syndrome				
Subjects at risk, n	6192	15,487	5096	257,721
Incident events, n (male/female)	143 (125/18)	420 (242/178)	59 (39/20)	5480 (3645/1835)
Person-years at risk, n	34,658	87,903	27,287	1,712,019
Overall incidence rates				
-Crude rates	4.1 (3.4–4.8)	4.8 (4.3–5.2)	2.2 (1.6–2.7)	3.2 (3.1–3.3)
-Standardized rates^a^	4.3 (3.4–5.2)	5.4 (4.8–5.9)	4.7 (3.1–6.2)	
Male incidence rates				
-Crude rates	5.3 (4.4–6.3)	6.3 (5.5–7.1)	3.3 (2.3–4.3)	4.4 (4.3–4.6)
-Standardized rates^b^	6.3 (5.1–7.5)	7.0 (6.1–8.0)	5.9 (3.7–8.0)	
Female incidence rates				
-Crude rates	1.6 (0.9–2.3)	3.6 (3.1–4.1)	1.3 (0.7–1.9)	2.1 (2.0–2.2)
-Standardized rates^b^	2.4 (1.0–3.7)	3.8 (3.2–4.4)	3.6 (1.4–5.8)	
Composite stroke^c^				
Subjects at risk, n	6247	15,536	5106	257,481
Incident events, n (male/female)	147 (110/37)	463 (219/244)	66 (31/35)	8001 (4405/3596)
Person-years at risk, n	35,017	88,075	27343	1,707,096
Overall incidence rates				
-Crude rates	4.2 (3.5–4.9)	5.3 (4.8–5.7)	2.4 (1.8–3.0)	4.7 (4.6–4.8)
-Standardized rates^a^	5.4 (4.3–6.6)	5.9 (5.4–6.5)	5.7 (3.9–7.4)	
Male incidence rates				
-Crude rates	4.6 (3.8–5.5)	5.6 (4.8–6.3)	2.6 (1.7–3.5)	5.3 (5.1–5.4)
-Standardized rates^b^	5.9 (4.7–7.1)	6.5 (5.6–7.3)	5.2 (2.9–7.4)	
Female incidence rates				
-Crude rates	3.3 (2.2–4.4)	5.0 (4.4–5.6)	2.3 (1.5–3.0)	4.1 (4.0–4.3)
-Standardized rates^b^	5.0 (3.0–6.9)	5.5 (4.7–6.2)	6.1 (3.4–8.8)	
Venous thromboembolism				
Subjects at risk, n	6333	15,716	5110	262,600
Incident events, n (male/female)	98 (66/32)	268 (95/173)	56 (26/30)	3925 (2038/1887)
Person-years at risk, n	35,575	89,525	27,363	1,745,481
Overall incidence rates				
-Crude rates	2.8 (2.2–3.3)	3.0 (2.6–3.4)	2.0 (1.5–2.6)	2.2 (2.2–2.3)
-Standardized rates^a^	3.6 (2.6–4.6)	3.2 (2.8–3.6)	3.5 (2.3–4.7)	
Male incidence rates				
-Crude rates	2.7 (2.1–3.4)	2.4 (1.9–2.8)	2.2 (1.3–3.0)	2.4 (2.3–2.5)
-Standardized rates^b^	3.0 (2.2–3.8)	2.6 (2.1–3.2)	3.1 (1.8–4.4)	
Female incidence rates				
-Crude rates	2.8 (1.9–3.8)	3.5 (3.0–4.0)	2.0 (1.3–2.7)	2.1 (2.0–2.2)
-Standardized rates^b^	4.2 (2.4–5.9)	3.7 (3.2–4.3)	3.9 (1.9–5.9)	

Rates are presented as number of events per 1000 person-years at risk. All rates are calculated with 95% confidence interval given in parenthesis

*ACS* acute coronary syndrome, *VTE* venous thromboembolism, *AS* ankylosing spondylitis, *PsA* psoriatic arthritis, *uSpA* undifferentiated spondyloarthritis, *GP* general population

^a^Age- and sex-adjusted with the general population (GP) cohort as reference

^b^Age-adjusted with the GP cohort as reference

^c^Composite stroke includes ischemic, hemorrhagic, unspecified stroke, and transient ischemic attack

### Stroke

**Fig. 2:**
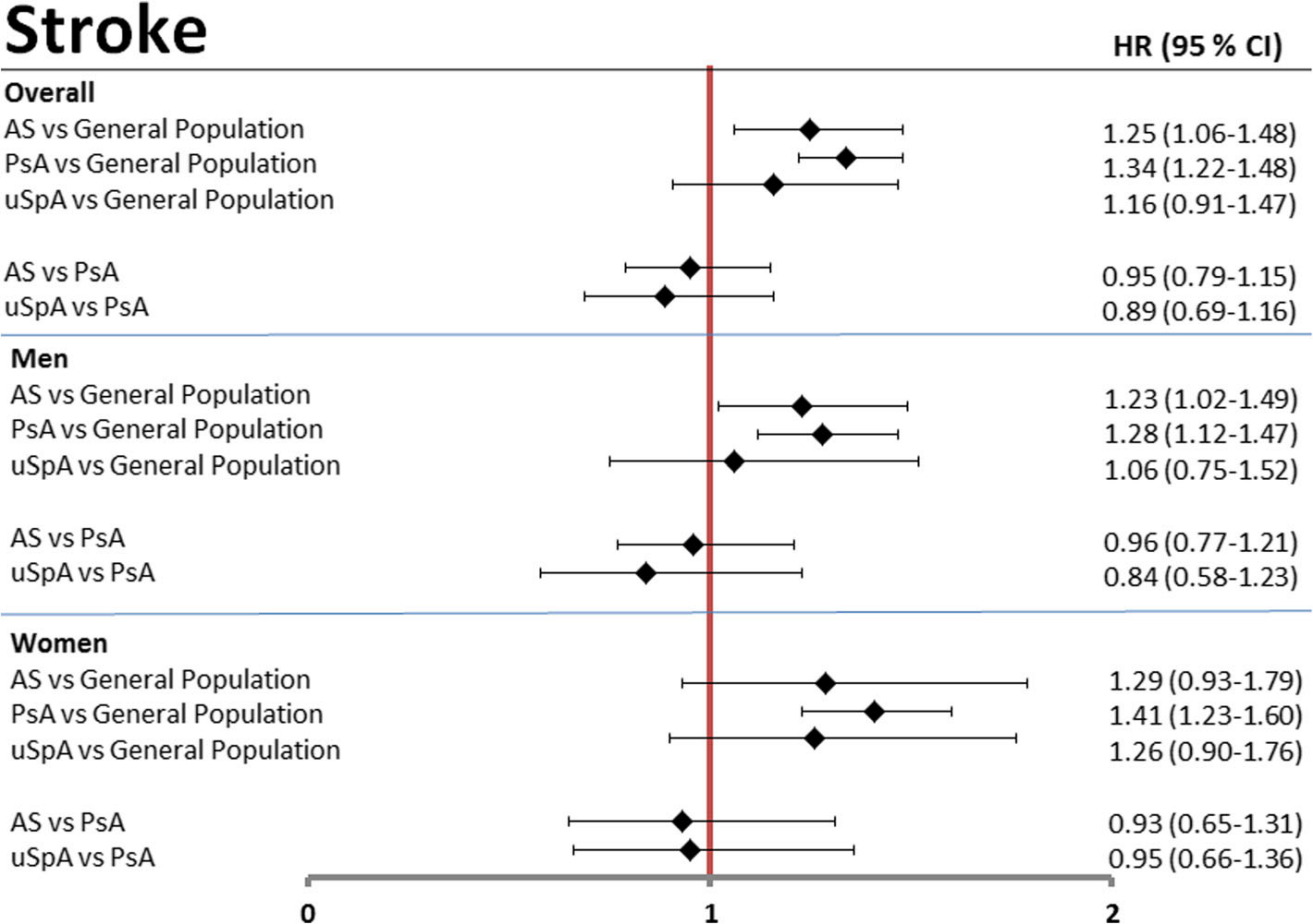
Age- and sex-adjusted hazard ratios (HRs) for composite stroke. Age- and sex-adjusted HRs, overall and stratified by sex, are presented with 95% confidence interval (CI) in patients with AS, PsA, and uSpA, using GP comparators and PsA patients as reference. *AS* ankylosing spondylitis, *PsA* psoriatic arthritis, *uSpA* undifferentiated spondyloarthritis

During follow-up, 147, 463, and 66 incident composite stroke events occurred in the AS, PsA, and uSpA cohorts, respectively, resulting in standardized incidence rates of 5.4, 5.9, and 5.7 stroke events per 1000 person-years at risk compared to 4.7 in the GP cohort (Table 2). The age- and sex-adjusted HRs were significantly increased in AS (1.25 (1.06–1.48)) and PsA (1.34 (1.22–1.48)) and non-significantly in uSpA (1.16 (0.91–1.47)) patients compared to the GP cohort. There were no significant differences between AS and uSpA compared to the PsA cohort (Fig. 2).

**Fig. 3:**
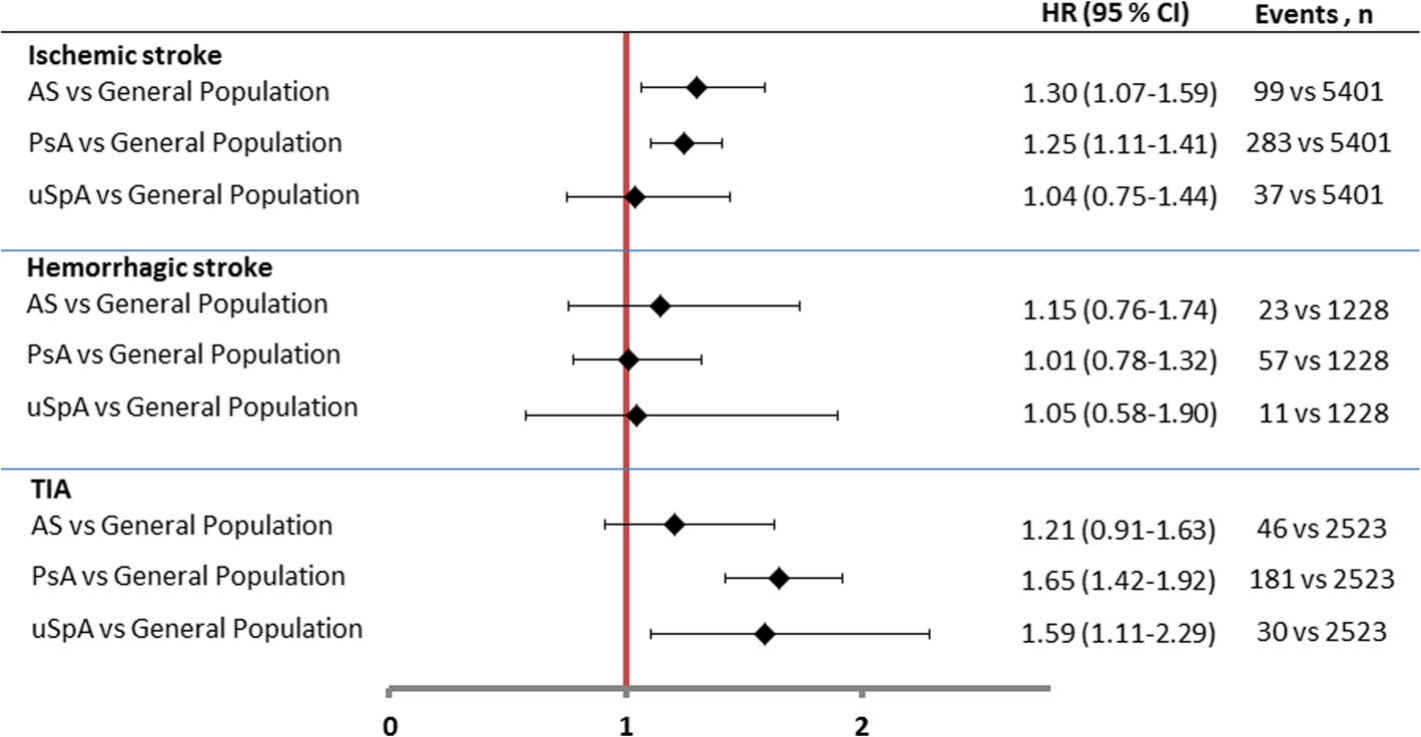
Age- and sex-adjusted HRs for ischemic stroke, hemorrhagic stroke and transient ischemic attack (TIA). Age- and sex-adjusted hazard ratios (HRs) are presented with 95% confidence interval (CI) in patients with AS, PsA, and uSpA, using GP comparators as reference. *AS* ankylosing spondylitis, *PsA* psoriatic arthritis, *uSpA* undifferentiated spondyloarthritis

The results for ischemic stroke were similar to those for stroke as a composite outcome. When TIA was used as an outcome, significantly increased HRs were noted in PsA and uSpA, and nonsignificantly in AS compared to GP. In contrast, no significant differences in HRs were noted for hemorrhagic stroke (Fig. 3).

### Venous thromboembolism

**Fig. 4:**
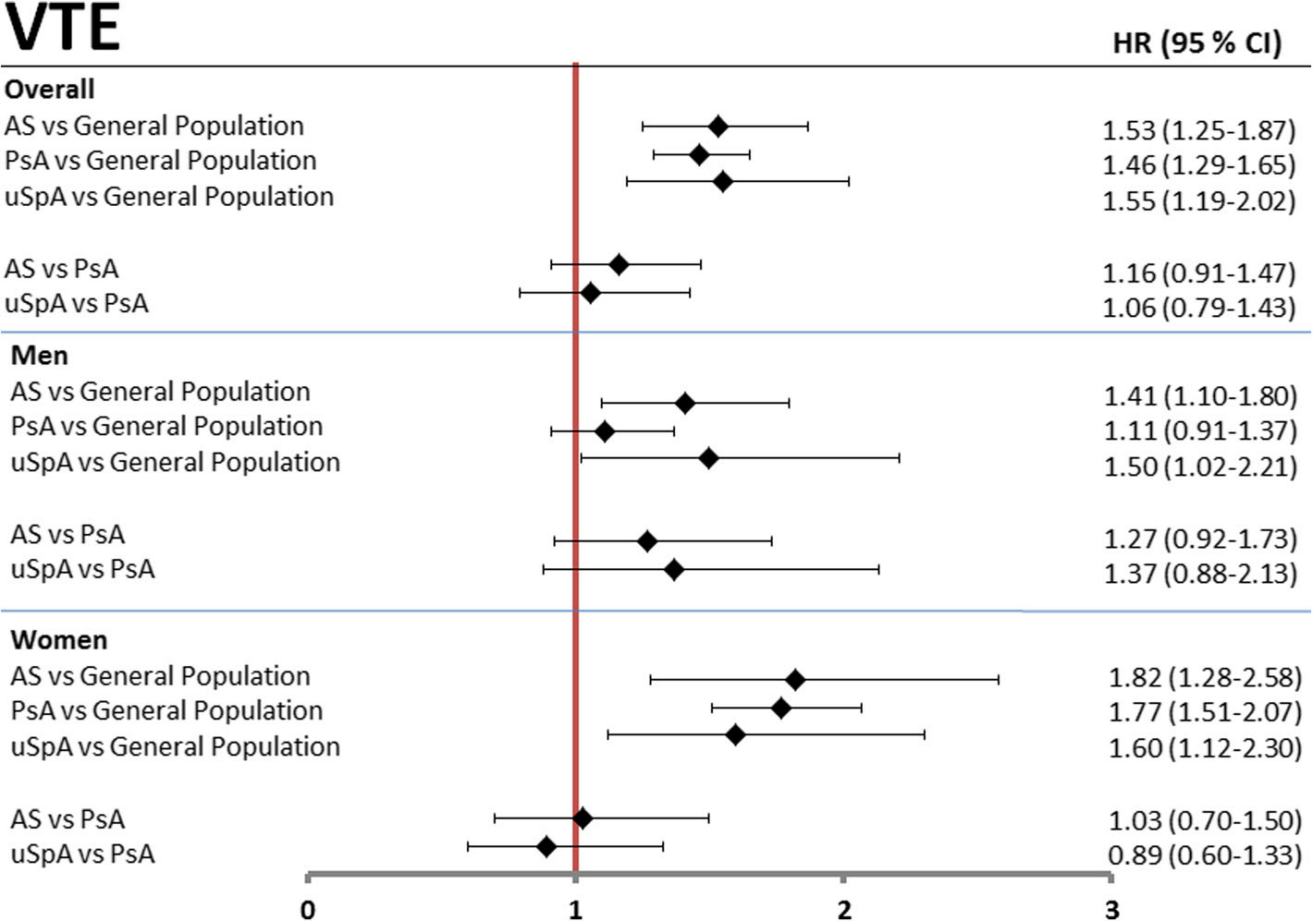
Age- and sex-adjusted hazard ratios (HRs) for venous thromboembolism (VTE). Age- and sex-adjusted HRs, overall and stratified by sex, are presented with 95% confidence interval (CI) in patients with AS, PsA, and uSpA, using GP comparators and PsA patients as reference. *AS* ankylosing spondylitis, *PsA* psoriatic arthritis, *uSpA* undifferentiated spondyloarthritis

Based on 98, 268 and 56 incident VTE in the AS, PsA, and uSpA cohort, respectively, the standardized incidence rates were 3.6, 3.2, and 3.5 VTE events per 1000 person-years at risk compared to 2.2 in the GP cohort (Table 2). The HRs were significantly increased by around 50% in all of the three SpA cohorts compared to the GP cohort, both overall and stratified by sex, with the exception of male PsA patients (Fig. 4).

#### Patients diagnosed with ≥ 2 subtypes of SpA

Patients (*n* = 1931) excluded prior to study entry or censored during follow-up based on ICD codes for two or more of the three different SpA subtypes were analyzed separately. These patients (55% men, mean age 45.2 years) were to a larger extent treated with DMARD (48.2%) and TNF inhibitors (20.1%) at baseline (Additional file 3). The majority of the patients (*n* = 1169) had an overlap of AS and uSpA diagnoses. Incidence rates and HRs for the cardiovascular outcomes are summarized in Additional file 3 and did not diverge substantially from the main results.

## Discussion

In this nationwide prospective population-based cohort study we found an increased risk of 36–76% for ACS and 50% for VTE in all subtypes of SpA compared to the general population. A less pronounced, but still increased risk was noted for stroke in AS and PsA, but not in uSpA. Female PsA patients had an almost doubled risk of having a first-time ACS compared to GP comparators as well as a significantly increased risk compared to female AS patients. Apart from this, no statistically significant differences in cardiovascular events were found between the SpA cohorts, although the PsA patients tended to have the highest risks for both stroke and ACS. The absolute risks of ACS, VTE, and stroke were modest in all cohorts, as would be expected considering the relatively low mean age of the cohorts.

In relation to other studies, our results in PsA are similar to the risk estimates for myocardial infarction found in severe psoriasis and somewhat lower than the risk estimates for stroke in severe psoriasis according to a meta-analysis from 2013 [11]. Two previous studies have investigated incident major adverse cardiovascular events (MACE), which include myocardial infarction and stroke, in PsA patients. We found a similar relative risk of stroke in PsA and a slightly higher relative risk of ACS/myocardial infarction compared to the British study using a primary health-care database to identify PsA patients and cardiovascular outcomes [17]. Li et al., using another British primary health-care database, only reports results of the composite outcome MACE and found an incidence rate ratio of 1.30 (95% CI 1.15–1.47) in PsA patients compared to patients without PsA [18]. Previous studies in AS have shown divergent results. Two prior studies, using different British primary health-care databases to identify patients with ankylosing spondylitis, did not find an increased risk of myocardial infarction or stroke [6, 7]. However, Keller et al. found a more than doubled risk of stroke in AS [4]. This is in contrast to our risk estimates, which are in agreement to Chou et al. who found an increased risk of ACS with HRs 1.36 (1.16–1.59) [5]. Possible explanations for these divergent results could be selection of patients and validity of outcome assessments. Our study is population-based, with case ascertainment based on diagnoses set by specialists in rheumatology or internal medicine. This together with the generally well-validated National Patient Register, and the large patient groups, support the accuracy of the present results [31].

Results regarding VTE risks in SpA patients are scarce. A register-based Danish study found an even higher risk increase of VTE in patients with severe psoriasis compared to our findings [12]. However, in our study male PsA patients, in contrast to the other SpA patients, did not have a significantly increased risk of VTE compared to GP. Increased risks of VTE have also been demonstrated in other chronic inflammatory diseases, such as RA and inflammatory bowel disease [32–36].

In the sub-analysis of different types of stroke, the relative risk estimates were similar for composite stroke, ischemic stroke and TIA, which is not surprising considering that the latter two constitute the majority of all strokes [37]. For the minor group of stroke diagnosed as hemorrhagic stroke no differences were seen when comparing the SpA cohorts to the GP cohort. The stroke subtypes share some risk factors, such as hypertension, whereas other risk factors are more associated with one or the other, such as vascular malformations with hemorrhagic stroke and atrial fibrillation with ischemic stroke and TIA [38, 39]. Previous studies have found an association between inflammatory markers and ischemic stroke specifically, which could explain the different risks for ischemic and hemorrhagic stroke found in our study of patients with SpA patients compared to the GP cohort [40–42].

We observed increased risks in SpA patients of both arterial and venous thromboembolic events, which traditionally are considered having different risk factor panorama. Established risk factors for VTE include major surgery, malignant disease as well as multiple trauma and fractures [43, 44]. Shared risk factors for venous and arterial thromboembolism, such as obesity, diabetes and smoking, have been suggested by some, but not all studies [45–50]. Furthermore, inflammation has been suggested to be involved in both the atherosclerosis and in the VTE process and also to constitute a possible link between them [44, 51].

We have deliberately avoided making adjustments for other factors than age and sex since the aim was to assess rather than to attribute any increased risks of cardiovascular events seen in the SpA cohorts compared to the GP cohort. Our aim was thus not to examine to which extent inflammation, specific disease manifestations of the different SpA diagnoses or traditional CVD and VTE risk factors contributed to this increased risk. Due to the register-based design we lack detailed information on an individual level, both with regard to traditional risk factors for CVD, such as smoking habits, physical activity, body mass index and hereditary factors, as well as markers of inflammation and disease activity. Residual confounding factors would thus have been very likely if we had adjusted only for comorbidities and pharmacological treatment. Furthermore, since the patients had a prevalent SpA diagnosis, any differences in baseline comorbidities and treatments could also have been a consequence of previous disease manifestations. However, the increased risk for ACS and stroke found in all SpA subtypes emphasizes the importance of identification of and intervention against cardiovascular risk factors in SpA patients. These findings also support the new EULAR recommendations for CVD risk management which include recommendation of CVD risk assessment at least once every 5 years for AS and PsA patients[2].

Some limitations to the study need to be acknowledged. First, we cannot exclude misclassifications of the SpA diagnoses or the cardiovascular outcomes. The ICD codes used for both identification of AS, PsA, and uSpA have been used in other Swedish register-based studies and AS, uSpA, PsA, ACS/myocardial infarction, ischemic and hemorrhagic stroke are validated before with high positive predictive values [29–32, 52–55]. We analyzed patients with more than one of the three studied SpA subtypes separately to avoid case mixing. Due to similarities in clinical presentation, some remaining overlapping between the SpA subtypes is probably inevitable. Second, patients with SpA solely followed in primary care, presumably patients with a less severe disease, are not identified, and this may influence the generalizability of our results. In Sweden SpA patients without DMARDs may be monitored in primary care, but are predominately diagnosed in rheumatology or internal medicine outpatient care at least once, and would thus have been identified in our study. Third, for some cardiovascular events, such as hemorrhagic stroke, the low number of events may have hampered the statistical power. In addition, we cannot exclude that the generally lower incidence rates of ACS found in women was a consequence of under-recognition of ischemic heart disease due to different clinical presentation than in men [56].

There are also several strengths to the present study. First, it is the first study to compare the risk of CVD events for the different subtypes of SpA in the same setting simultaneously and also reporting CVD outcomes in the relatively large group classified as uSpA. The uSpA is a less clearly defined subtype of SpA, where previous research about CVD risks is especially lacking. Second, it is a population-based study of health-care registers, minimizing problems with selection bias. Third, due to the register-based approach there is negligible loss of follow-up.

## Conclusions

Patients with AS, PsA, and uSpA are at increased risk for ACS and stroke, which emphasizes the importance of identification of and intervention against cardiovascular risk factors in SpA patients. These findings also support the new EULAR recommendations for CVD risk management which include recommendation of CVD risk assessment at least once every 5 years for AS and PsA patients [2]. Increased alertness for VTE is warranted in patients with SpA.

## Additional files

Additional file 1: List of ICD and ATC codes used to identify patients, cardiovascular events, baseline comorbidities, and pharmacological treatment. (DOCX 21 kb)

Additional file 2: Age- and sex-standardized prevalence and corresponding prevalence ratios (PRs) for prior ACS, stroke, and VTE for each SpA cohort, using the age and sex distribution in the GP cohort as standard/reference. (DOCX 12 kb)

Additional file 3: Baseline characteristics, incidence rates and HRs of ACS, stroke, and VTE in patients diagnosed with ≥ 2 subtypes of SpA. (DOCX 20 kb)

## Abbreviations

ACS: Acute coronary syndrome
AS: Ankylosing spondylitis
ATC: Anatomical Therapeutic Chemical Classification
bDMARDs: Biological disease-modifying antirheumatic drugs
CI: Confidence interval
COPD: Chronic obstructive pulmonary disease
CVD: Cardiovascular disease
DMARD(s): Disease-modifying antirheumatic drug(s)
EULAR: European League Against Rheumatism
GP: General population
HRs: Hazard ratios
ICD: International Classification of Diseases
MACE: Major adverse cardiovascular events
NPR: National Patient Register
NSAIDs: Nonsteroidal anti-inflammatory drugs
PDR: Prescribed Drug Register
PPVs: Positive predictive values
PRs: Prevalence ratios
PsA: Psoriatic arthritis
RA: Rheumatoid arthritis
SD: Standard deviation
SIRs: Standardized incidence rates
SpA: Spondyloarthritis
SRQ: Swedish Rheumatology Quality register
TIA: Transient ischemic attack
uSpA: Undifferentiated spondyloarthritis
VTE: Venous thromboembolism
