# Supplementary material for: Are ankylosing spondylitis, psoriatic arthritis and undifferentiated spondyloarthritis associated with an increased risk of cardiovascular events? A prospective nationwide population-based cohort study
Source: Arthritis Res Ther. 2017 May 18;19:102. doi: 10.1186/s13075-017-1315-z (PMC5437558; doi:10.1186/s13075-017-1315-z)
Supplement: Supplementary file 1 — List of ICD and ATC codes used to identify patients, cardiovascular events, baseline comorbidities, and pharmacological treatment. (DOCX 21 kb) [file 13075_2017_1315_MOESM1_ESM.docx]

**Table S1. ICD- and ATC- codes used to identify patients, cardiovascular events, baseline comorbidities and pharmacological treatment.**

| **National Patient Register**  **(NPR)** | **ICD 10**  1997- | **ICD 9**  1987-1996 | **ICD 8**  1968-1986 | **ICD 7**  1964-1968 |
| --- | --- | --- | --- | --- |
| **Rheumatic diagnoses** |  |  |  |  |
| Ankylosing spondylitis | M45 | 720A | 712.40 | - |
| Psoriatic arthritis | L40.5, M07.0-3 | 696A, 713D | 696.00 | - |
| Undifferentiated SpA | M46.8/9 | 720B/C/W/X | 713.13, 726.99 | - |
| Rheumatoid arthritis | M05, M06.0/2/3/ 8/9, M12.3 | - | - | - |
| SLE | M32.0/1/8/9 | - | - | - |
| **Cardiovascular events** |  |  |  |  |
| Acute coronary syndrome | I20.0, I21 | 410, 411B | 410.00/07/97/99 | 420.10/17/18 |
| Composite stroke | I60-61, I63-64, G45 | 430-431, 433-436 | 430, 431.00/08/09/  90/98/99, 432-436 | 330, 331.00-09, 332.00-19, 332.29, 333.99. 334.00/09 |
| Ischemic stroke | I63 | 433-434 | 432-434 | 332.00-19, 332.29 |
| Hemorrhagic stroke | I60-61 | 430-431 | 430, 431.00/08/09/ 90/98/99 | 330, 331.00-09 |
| Transient ischemic attack | G45 | 435 | 435 | 333.99. 334.00 |
| Venous thromboembolism | I26, I80.1/2, I81, I82.2/3/8/9 | 415B, 451B, 452, 453C/D/W/X | 450-452 | 463-466 |
| **SpA-related comorbidities** |  |  |  |  |
| Anterior uveitis | H20, H221 | 364A-364B | - | - |
| Inflammatory bowel disease | K50-K51 | 555-556 | 563.00/10, 569.02 | - |
| Psoriasis | L40 | 696 | 696 | - |
| **Other comorbidities** |  |  |  |  |
| Ischemic heart disease | I20-I25 | 410-414 | 410-414 | - |
| Diabetes | E10-E14, O24 | 250, 648A | 250 | - |
| COPD | J41-J44 | 491-492, 496 | 490, 491.01/2/4, | - |
| Atrial fibrillation or flutter | I48 | 427D | 427.92 | 433.12/13 |
| **Other atherosclerotic disease** | **G45, I65-I66, I67.2, I69, I70-I72, I73.1, I73.9, I74, K55.0-K55.1** | 433-435, 437A-437B, 438, 440-442, 443B, 443X, 444, 557A-557B | 432, 435, 437, 440-442, 443.10, 443.9, 444, 445.0 |  |
| **Dispensed prescription in Prescribed Drug Register (PDR)** | **ATC** | | | |
| Any DMARD | A07EC01, L04AA15, L04AD01, L04AX01, L01BA01, L04AX03, M01CB01, M01CB03, P01BA01, P01BA02, L01XC02, L04AA24, L04AB01/2/4/5/6, L04AC03, L04AC07 | | | |
| - TNF-inhibitors | L04AB01/2/4/5/6 | | | |
| - Methotrexate | L01BA01, L04AX03 | | | |
| - Sulfasalazin | A07EC01 | | | |
| NSAIDs | M01AB01/05/55/15, M01AC01/2/5/6, M01AE01/51/02/03/09/14/17, M01AH01/3/4/5, M01AX01 | | | |
| Prednisone | H02AB06 | | | |
| Oral anti-diabetics or insulin | A10A/B | | | |
| Anti-hypertensive | C02A, C02CA, C03A/B/C/D/E, C07A/F, C08, C09 | | | |
| Statins | C10AA | | | |
| Aspirin | B01AC06/30/56 | | | |
| Warfarin | B01AA03 | | | |

ICD, International Classification of Diseases; ATC, Anatomical Therapeutic Chemical Classification; SpA, spondyloarthritis; COPD, chronic obstructive pulmonary disease; DMARD, disease-modifying anti-rheumatic drug; NSAIDs, nonsteroidal anti-inflammatory drugs
