# Supplementary material for: Are ankylosing spondylitis, psoriatic arthritis and undifferentiated spondyloarthritis associated with an increased risk of cardiovascular events? A prospective nationwide population-based cohort study
Source: Arthritis Res Ther. 2017 May 18;19:102. doi: 10.1186/s13075-017-1315-z (PMC5437558; doi:10.1186/s13075-017-1315-z)
Supplement: Supplementary file 2 — Age- and sex-standardized prevalence and corresponding prevalence ratios (PRs) for prior ACS, stroke, and VTE for each SpA cohort, using the age and sex distribution in the GP cohort as standard/reference. (DOCX 12 kb) [file 13075_2017_1315_MOESM2_ESM.docx]

**Table S2.** Age- and sex-standardized prevalence and corresponding prevalence ratios (PRs) at start of follow-up

|  | **Ankylosing spondylitis** | | | **Psoriatic arthritis** | | | **Undifferentiated SpA** | | |
| --- | --- | --- | --- | --- | --- | --- | --- | --- | --- |
| Covariates | Prevalence* | PR** | 95 % CI | Prevalence* | PR** | 95 % CI | Prevalence* | PR** | 95 % CI |
| **Prior ACS** | 4.6 | 1.4 | 1.3-1.6 | 4.2 | 1.3 | 1.2-1.4 | 4.3 | 1.3 | 1.1-1.5 |
| **Prior stroke** | 4.3 | 1.3 | 1.1-1.4 | 3.8 | 1.1 | 1.0-1.2 | 4.5 | 1.3 | 1.2-1.5 |
| **Prior VTE** | 2.3 | 1.6 | 1.4-1.9 | 2.2 | 1.5 | 1.4-1.7 | 3.0 | 2.1 | 1.8-2.4 |

*****Age- and sex-standardized prevalence (%) at start of follow-up, using the age- and sex-distribution in the general population (GP) cohort as standard.

**Prevalence ratio (PR) using the age- and sex-standardized prevalence in the spondyloarthritis (SpA) cohorts and the crude prevalence in the GP cohort (reference).
