# Supplementary material for: Are ankylosing spondylitis, psoriatic arthritis and undifferentiated spondyloarthritis associated with an increased risk of cardiovascular events? A prospective nationwide population-based cohort study
Source: Arthritis Res Ther. 2017 May 18;19:102. doi: 10.1186/s13075-017-1315-z (PMC5437558; doi:10.1186/s13075-017-1315-z)
Supplement: Supplementary file 3 — Baseline characteristics, incidence rates and HRs of ACS, stroke, and VTE in patients diagnosed with ≥ 2 subtypes of SpA. (DOCX 20 kb) [file 13075_2017_1315_MOESM3_ESM.docx]

**Table S3. Baseline characteristics of patients with mixed SpA**

|  | **Mixed SpA (n=1931)** |
| --- | --- |
| **Sex** |  |
| Males | 1062 (55.0) |
| Females | 869 (45.0) |
| **Age (years)** |  |
| Mean (SD) | 45.2 (12.6) |
| 18-29 years | 207 (10.7) |
| 30-39 years | 494 (25.6) |
| 40-49 years | 535 (27.7) |
| 50-59 years | 390 (20.2) |
| 60-69 years | 242 (12.5) |
| 70-79 years | 59 (3.1) |
| ≥80 years | 4 (0.2) |
| **Start of follow-up** |  |
| 2006-2007 | 1265 (65.5) |
| 2008-2010 | 666 (34.5) |
| **Level of education**   - ≤9 years - 10-12 years - >12 years - Missing | 310 (16.1)  964 (49.9)  643 (33.3)  14 (0.7) |
| **SpA-related comorbidities*** |  |
| Anterior uveitis | 382 (19.8) |
| Inflammatory bowel disease | 135 (7.0) |
| Psoriasis | 756 (39.2) |
| **SpA-related medications**** |  |
| Any DMARD | 930 (48.2) |
| - TNF-inhibitors | 388 (20.1) |
| - Methotrexate | 432 (22.4) |
| - Sulfasalazin | 332 (17.2) |
| NSAIDs | 1066 (55.2) |
| Prednisone | 294 (15.2) |
| **Other comorbidities*** |  |
| Ischemic heart disease   - Acute coronary syndrome | 70 (3.6)  41 (2.1) |
| Stroke composite | 28 (1.5) |
| - Ischemic stroke | 11 (0.6) |
| - Hemorrhagic stroke | 3 (0.2) |
| - Transient ischemic attack | 14 (0.7) |
| Venous thromboembolism | 33 (1.7) |
| Diabetes | 54 (2.8) |
| COPD | 26 (1.3) |
| Atrial fibrillation or flutter | 33 (1.7) |
| Other atherosclerotic disease | 33 (1.7) |
| **Dispensed prescriptions**** |  |
| Oral anti-diabetics or insulin | 48 (2.5) |
| Anti-hypertensive | 390 (20.2) |
| Statins | 127 (6.6) |
| Aspirin | 83 (4.3) |
| Warfarin | 19 (1.0) |

Mixed spondyloarthritis (SpA) includes the patients diagnosed with ≥ 2 different SpA subtypes before start of follow-up or during follow-up (censored). Age are given in mean (SD). All other data are presented in number (%).* Prevalent comorbidity at baseline, defined by identification of specified ICD-codes in the National Patient Register prior to start of follow-up. ** Dispensed prescription in Prescribed Drug Register or intravenous bDMARD in Swedish Rheumatology Quality register within 6 months prior to start of follow-up. DMARD, disease-modifying anti-rheumatic drug; NSAIDs, nonsteroidal anti-inflammatory drugs; COPD, chronic obstructive pulmonary disease.

**Table S4. Incidence rates and hazard ratios of ACS, stroke and VTE in mixed SpA patients.**

|  | **Mixed SpA** |
| --- | --- |
| **ACUTE CORONARY SYNDROME** |  |
| Subjects at risk, n | 1890 |
| Incident events, n (male/female) | 21 (17/4) |
| Person-years at risk, n | 10320 |
| **Overall incidence rates and HRs** |  |
| -Crude rates | 2.0 (1.2-2.9) |
| -Standardized rates* | 4.2 (1.7-6.6) |
| -HRs, GP as reference | 1.20 (0.78-1.85) |
| -HRs, PsA as reference | 0.70 (0.45-1.10) |
| **Male incidence rates and HRs** |  |
| -Crude rates | 3.0 (1.6-4.5) |
| -Standardized rates** | 7.4 (2.4-12.3) |
| -HRs, GP as reference | 1.24 (0.77-2.00) |
| -HRs, PsA as reference | 0.80 (0.49-1.31) |
| **Female incidence rates and HRs** |  |
| -Crude rates | 0.9 (0.0-1.7) |
| -Standardized rates** | 1.2 (0.0-2.3) |
| -HRs, GP as reference | 1.02 (0.38-2.73) |
| -HRs, PsA as reference | 0.47 (0.17-1.28) |
|  |  |
| **COMPOSITE STROKE#** |  |
| Subjects at risk, n | 1903 |
| Incident events, n (male/female) | 30 (15/15) |
| Person-years at risk, n | 10368 |
| **Overall incidence rates and HRs** |  |
| -Crude rates | 2.9 (1.9-3.9) |
| -Standardized rates* | 8.2 (2.9-13.6) |
| -HRs, GP as reference | 1.39 (0.97-1.99) |
| -HRs, PsA as reference | 1.07 (0.74-1.56) |
| **Male incidence rates and HRs** |  |
| -Crude rates | 2.6 (1.3-4.0) |
| -Standardized rates** | 3.6 (1.6-5.5) |
| -HRs, GP as reference | 1.06 (0.64-1.76) |
| -HRs, PsA as reference | 0.83 (0.49-1.41) |
| **Female incidence rates and HRs** |  |
| -Crude rates | 3.2 (1.6-4.8) |
| -Standardized rates** | 12.7 (2.5-23.0) |
| -HRs, GP as reference | **2.01 (1.21-3.34)** |
| -HRs, PsA as reference | 1.49 (0.88-2.54) |
|  |  |
| **VENOUS THROMBOEMBOLISM** |  |
| Subjects at risk, n | 1898 |
| Incident events, n (male/female) | 19 (8/11) |
| Person-years at risk, n | 10358 |
| **Overall incidence rates and HRs** |  |
| -Crude rates | 1.8 (1.0-2.7) |
| -Standardized rates* | 2.1 (0.9-3.3) |
| -HRs, GP as reference | 1.39 (0.88-2.18) |
| -HRs, PsA as reference | 0.98 (0.61-1.58) |
| **Male incidence rates and HRs** |  |
| -Crude rates | 1.4 (0.4-2.4) |
| -Standardized rates** | 1.5 (0.4-2.7) |
| -HRs, GP as reference | 0.95 (0.48-1.91) |
| -HRs, PsA as reference | 0.86 (0.42-1.79) |
| **Female incidence rates and HRs** |  |
| -Crude rates | 2.4 (1.0-3.8) |
| -Standardized rates** | 2.7 (0.6-4.7) |
| -HRs, GP as reference | **2.05 (1.13-3.72)** |
| -HRs, PsA as reference | 1.09 (0.59-2.03) |

Incidence rates are presented as number of events per 1000 person-years at risk and with 95 % confidence interval (CI) in parenthesis. Age- and sex-adjusted hazard ratios (HRs), overall and stratified by sex, are presented with 95% confidence interval (CI), using general population (GP) comparators and psoriatic arthritis (PsA) patients as reference. *Age- and sex-adjusted with the GP cohort as reference. **Age-adjusted with the GP cohort as reference. # Composite stroke includes ischemic, hemorrhagic, unspecified stroke and transient ischemic attack.
